# Effects of nitrogen fertilizer on protein accumulation in basal-middle and apical kernels of different low nitrogen tolerant maize hybrids

**DOI:** 10.3389/fpls.2025.1526026

**Published:** 2025-02-21

**Authors:** Pi-Jiang Yin, Xing-Long Wang, Ya-Wei Wu, Fan Liu, Ye Tao, Qin-Lin Liu, Tian-Qiong Lan, Dong-Ju Feng, Fan-Lei Kong, Ji-Chao Yuan

**Affiliations:** ^1^ College of Agronomy, Sichuan Agricultural University, Chengdu, China; ^2^ Key Laboratory of Crop Ecophysiology and Farming System in Southwest China, Ministry of Agriculture, Chengdu, China; ^3^ Crop Ecophysiology and Cultivation Key Laboratory of Sichuan Province, Chengdu, China; ^4^ School of Life Science and Engineering, Southwest University of Science and Technology, Mianyang, China

**Keywords:** nitrogen fertilizer, protein, protein components, growth model, basalmiddle kernel, apical kernel, grain yield, maize

## Abstract

Selecting low-nitrogen(N)-tolerant maize hybrids represent an effective approach to enhancing nitrogen use efficiency grain yield. However, the impact of nitrogen fertilization on protein accumulation in low-N-tolerant hybrids remain insufficiently explored. In this paper, a two-year field orientation trial was conducted at four nitrogen fertilizer rate with the different low-N-tolerant maize hybrids. The effect of nitrogen fertilization on the accumulation of protein and its fractions different kernels positions of different low-N-tolerant maize hybrids was studied. The results showed that the protein yield of ZH311 maize kernels was significantly higher than that of XY508, especially under low-N conditions (0N and 150N), and was 25.7%-36.2% higher than that of XY508. There was a significant correlation between protein yield and the accumulation of crude protein and protein fractions. Compared with XY508, the crude protein of ZH311 entered the rapid growth stage later and lasted for a relatively shorter period, but it was 50.8%-53.0% higher due to its higher accumulation rates (v_2_ and v_3_) in its middle and late stages, especially in the apical grains. Under low-N conditions, the difference in crude protein accumulation between the apical and basal-middle kernels of ZH311 was only 4.3-8.2%, whereas the difference in XY508 was 29.9-37.3%, suggesting that low-N-tolerant maize hybrids improve protein yield by increasing the accumulation of proteins and their fractions in the apical kernels. Nitrogen fertilization had a greater effect on protein accumulation and yield in XY508, especially on the top kernel and protein yield. In the future, more attention should be paid to the effect of apical kernels when breeding high-quality maize hybrids tolerant to low nitrogen.

## Highlights

- Developed an accumulation model for protein and its fractions in maize grains, clarifying its main parameters;
- Apical grains protein is higher in low-N-tolerant hybrids and differs less from basal-middle;
- Nitrogen fertilizer more effectively increases protein yield in N-sensitive hybrids.

## 1 Introduction

Maize (*Zea mays* L.) is one of the most important grain crops in the world, a fundamental food source for humans and livestock, and is also extensively employed in industrial processing and energy production (Ning et al., 2024). With a growing global population (expected to be 9.5 billion in 2050) (Food and Agriculture Organization (FAO), 2019), climate change, and increasing demand for food, the yield and quality of maize have a direct impact on global food security and economic stability (Tilman et al., 2011, Fukase and Martin, 2020). Agronomists have traditionally prioritized improving maize yield, and a few recent studies have been done on the quality of silage maize and sweet waxy maize (Lu et al., 2015; Zhao et al., 2022). However, research on the formation of quality in seed maize, particularly in terms of protein quality, has lagged.

Protein is the second-largest storage fractions in maize grains, one of critical determinant of quality (Sethi et al., 2021), playing an essential role in both human and animal health. Maize grain proteins is mainly composed of four major fractions: albumin, globulin, prolamin, and glutelin (Nuss and Sherry, 2010). Albumin and globulin are classified as cytoplasmic proteins, while prolamin and glutelin function as storage proteins. Prior research has identified prolamin as one of key determinant of grain weight, yield, and nutritional quality (Tsai et al., 1978; Zhang et al., 2005), with emerging applications in pharmaceuticals and nutritional products (Shah et al., 2016). The content of protein and its fraction in mature maize grains exhibited significant variation across different hybrids (Liu et al., 2024), further influenced by environmental conditions and agronomic practices, including fertilization (Li and Li, 1995; Liu et al., 2022; Liu, 2022). However, comprehensive studies on the formation dynamics of protein especially the individual protein fractions, and their genotype-environment effects, remain limited. Maize grain weight varies significantly according to its position on the ear. The basal-middle grains inseminating earlier and receiving a preferential nutrient supply were fuller and heavier which are usually called superior grains (Shen et al., 2023), while the apical grains, called inferior grains, were smaller which limited its yield potential (Shen et al., 2016). Research has indicated that increasing the grain weight of apical grains and reducing the weight disparity between basal-middle and apical grains could improve grain yield (Liu et al., 2024). However, there are still few studies on the differences in the accumulation of protein and its fraction between basal-middle and apical grains, and it remains to be further investigated whether narrowing the differences in protein accumulation between superior and inferior grains can also improve protein yield.

Nitrogen is a critical nutrient for plant growth and development, and its optimal application can stimulate maize growth, enhance photosynthesis, and ultimately boost yield (Ladha et al., 2016; Mueller and Vyn, 2016). Nitrogen is also a core fraction of proteins, which generally contain 16% nitrogen (Nicolette and Hettie, 2013). Increased nitrogen fertilization not only provides essential elements for protein synthesis but also enhances nitrogen metabolism in maize grains by activating key metabolic enzymes such as glutamine synthetase (GS) and glutamate synthase (GOGAT), thereby resulting in an increased protein content (Zhu et al., 2017; Ochieng’ et al., 2021). However, some studies suggested that excessive nitrogen application might reduce protein content Zhao (2012). The application of nitrogen is the simplest and most effective measure to increase maize yield (Ladha et al., 2016; Mueller and Vyn, 2016). In many regions in China, excessive nitrogen application to boost maize yield has led to a decline in maize quality, environmental pollution, increased production costs, and reduced nitrogen use efficiency (Xu et al., 2018; Du et al., 2021; Yin et al., 2021; Xu et al., 2018). An important challenge in these areas is to moderately reduce nitrogen usage while maintaining high maize yield and quality, thus improving economic efficiency and reducing environmental damage. Previous studies have shown that using low-N-tolerant (low-N high efficient) maize hybrids is an effective technical approach (Li et al., 2020; Wu et al., 2022).

Different maize hybrids exhibit significant differences in nitrogen use efficiency and tolerance to low-N conditions (Wu et al., 2021). Low-N-tolerant hybrids could sustain high yields under low-N conditions, whereas low-N-sensitive hybrids require higher nitrogen inputs to achieve similar yields (Shen et al., 2016). Prior research has largely focused on root morphology, leaf photosynthesis, dry matter accumulation, grain filling characteristics, and yield formation of different low-N-tolerant maize hybrids, as well as their responses to nitrogen fertilization (Wu et al., 2019, 2021, 2022; Wu et al., 2022; Liu et al., 2024). However, little attention has been given to their dynamics of protein accumulation and the formation of its fraction.

We hypothesized that there were differences in protein accumulation between basal-middle and apical grains, as well as their response to nitrogen fertilization, in different low-nitrogen-tolerant maize hybrids. To this end, we conducted a two-year field experiment using the low-N-tolerant maize hybrid ZH311 and low-N-sensitive hybrid XY508. The objectives were: (1) to investigate the accumulation dynamics and key parameters of protein and its fractions in maize grains; (2) to analyze the differences of protein accumulation in different grain positions across low-N-tolerant maize hybrids; and (3) to explore the effects of nitrogen fertilizer on the accumulation of protein and its fractions in different low-N-tolerant maize hybrids. The findings from this study might offer critical insights for breeding high-protein maize hybrids and developing management strategies increasing grain yield quality.

## 2 Materials and methods

### 2.1 Experimental site and materials

During the 2017-2018 maize growing season, a two-year *in situ* field experiment was conducted in Zhongjiang County, Deyang City, Sichuan Province, China (31.03°N, 104.68°E). The climatic data for the experiment period is presented in **Supplementary Figure 1**. Before sowing, soil samples were collected from the 0-20 cm soil layer using the diagonal sampling method, with its organic matter, total nitrogen, alkaline nitrogen, available phosphorus, available potassium, and pH values measured in **Supplementary Table 1**. The experimental materials were the previously screened low-N-tolerant maize hybrid ZhengHong 311 (ZH311) and the low-N-sensitive maize hybrid XianYu 508 (XY508) (Wu et al., 2022; Liu et al., 2024). Seeds were provided by Sichuan Zhenghong Biotechnology Co. and Tieling Pioneer Seed Research Co., respectively.

### 2.2 Experimental design

A two-factor randomized block design was employed for maize hybrid and nitrogen fertilizer rate with three replications and a plot area of 42 m² (6 m × 7 m). The nitrogen rate was set at four levels of 0 kg N ha⁻¹ (0N), 150 kg N ha⁻¹ (150N), 300 kg N ha⁻¹ (300N), and 450 kg N ha⁻¹ (450N). Among these:300 kg ha^-1^ represents the normal N level, which is the customary N rate applied by local farmers, while 150 kg ha^-1^ and 450 kg ha^-1^ represent low and excess N level, respectively. Maize was sown on March 30, 2017, and April 6, 2018, respectively. The planting density were 52,500 plants per hectare with wide row of 1.1 m and narrow row of 0.5 m. The fertilizer was urea (46% nitrogen content), 50% as a base fertilizer applied at sowing and 50% as ear fertilizer applied at the 13-leaf stage, with additional 72 kg P₂O₅ ha⁻¹ and 90 kg K₂O ha⁻¹ as base fertilizers for each treatment. The base fertilizer was placed in a ditched dug between two narrow rows, while the ear fertilizer was applied in a hole dug near the maize plants. Other managements were the same as of local production practice.

### 2.3 Sampling and measurements

At the silking stage, 100 representative maize plants were carefully marked in each experimental plot. Every five days after silking, in each plot of each variety, five spikes were collected from marked plants at 5-day intervals and threshed manually. The grains from the upper one-third of the ear were pooled as apical grains, while those from the lower two-thirds were classified as basal-middle grains (Wu et al., 2022). The harvested grains were dried to a constant weight in an oven set at 60°C, finely ground, and then sieved through an 80-mesh (0.2 mm) sieve. The soluble protein content was quantified using the Coomassie Brilliant Blue assay method (Sedmak and Grossberg, 1977). Protein fractions were analyzed through the sequential extraction technique, while crude protein content was determined by the Kjeldahl method (Mehak et al., 2021).

### 2.4 Statistical analysis

Data were subjected to variance and regression analyses using SPSS (ver.27; IBM Corporation, USA, www.spss.com.cn) software, with mean comparisons conducted via the least significant difference (LSD₀.₀₅) method. Graphs were generated using Originpro (ver.2025; OriginLab Corporation, USA, https://www.originlab.com/), while correlation analysis, path analysis, variance partition analysis (VPA), and structural equation modelling (SEM) were performed in R version 4.2.2 (R Core Team, 2022). The post-silking dynamic changes of soluble protein content were modelled using a quadratic function, while the accumulation dynamics of protein and its fractions were fitted to a Logistic equation (Zhan et al., 2022):


(1) $$\text{y}=\text{a}/\left(1+\text{b}e^{-kt}\right)$$

Where, y denotes the accumulation amount of crude protein or its fractions within the grain, t represents the number of days after silking, a refers to the theoretical maximum value of y, and b and k are constants. The key parameters were calculated using the fitted equation, including the start time (t₁​) and end time (t₂) of the rapid increase of y, the final stop time (t₃), the initial–increase phase (T₁​), the fast–increase phase (T₂​), and the slight–increase phase (T₃), as well as the average increasing rates for each phase.


(2) $${\text{t}}_{1}=-\frac{1}{\text{k}}\text{ln}\frac{2+\sqrt{3}}{\text{b}},\;\;\;{\text{T}}_{1}={\text{t}}_{1}$$

(3) $${\text{t}}_{2}-\frac{1}{\text{k}}\text{ln}\frac{2-\sqrt{3}}{\text{b}},\;\;\;{\text{T}}_{2}={\text{t}}_{2}-{\text{t}}_{1}$$

(4) $${\text{t}}_{3}\text{=(lnb}+4.595）/\text{k,}\;\;\;{\text{T}}_{3}={\text{t}}_{3}-{\text{t}}_{2}$$

(5) $${\text{y}}_{i}=\text{a}\left(1={\text{be}}^{-kti}\right)$$

(6) $${\text{v}}_{1}={\text{y}}_{1}/{\text{T}}_{1}，{\text{v}}_{2}=\left({\text{y}}_{2}-{\text{y}}_{1}\right)/{\text{T}}_{2}，{\text{v}}_{3}=\left({\text{y}}_{3}-{\text{y}}_{2}\right)/{\text{T}}_{3}$$

## 3 Results

### 3.1 Effects of nitrogen fertilizer rate on protein yield of different low-N-tolerant maize hybrids

**Figure 1:**
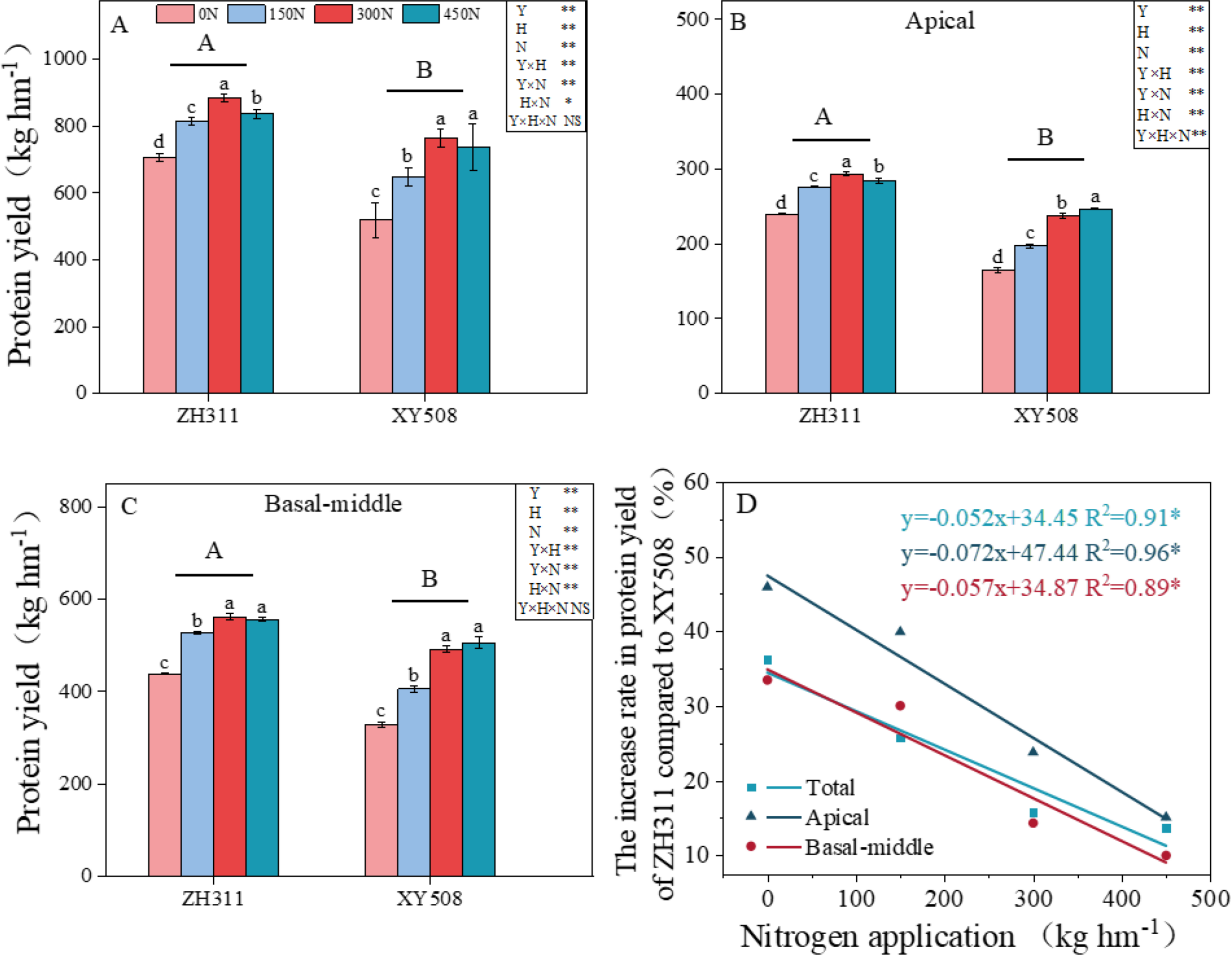
Effects of nitrogen fertilizer rate on protein yield of different low-N-tolerant maize hybrids (average from 2017-2018). **(A)** showed total protein yield, **(B)** showed apical grain protein yield, **(C)** showed basal-middle grain protein yield, and **(D)** showed the margin by which ZH311 protein yield exceeds that of XY508. The values represent mean ± SE. (n = 6). Different lowercase letters indicate significant differences between nitrogen treatments within the same hybrid at the P<0.05 level and different uppercase letters indicate significant differences between hybrids at the P<0.05 level. The symbol *, indicated significant at P<0.05. **, significant at P<0.01. NS, not significant at P>0.05.

The application of nitrogen fertilizer significantly enhanced grain protein yield by augmenting nitrogen content within the grains (**Supplementary Table 1**) and stimulating grain filling (**Figure 1**), with the effect being particularly pronounced in XY508. Compared to 0N, the protein yield of ZH311 increased by 15.4% to 25.2% under nitrogen applications ranging from 150 to 450 kg ha⁻¹, whereas XY508 exhibited an increase of 24.9% to 47.2% (**Figure 1A**). ZH311 demonstrated significantly higher protein yield than XY508, especially under low-N conditions (0N and 150N) (**Figure 1**). With each additional 100 kg ha⁻¹ of nitrogen fertilizer, the difference in protein yield between ZH311 and XY508 in apical and basal-middle grains decreased by 7.2 and 5.7 percentage points, respectively (**Figure 1D**). This suggests that the impact of increasing nitrogen fertilizer on enhancing protein yield is more substantial in XY508 grains compared to ZH311, particularly for apical grains.

The observed increase in protein yield is closely associated with grain weight, grain number per ear, and protein content (**Supplementary Table 2**). Correlation and path analysis revealed that grain weight, grain number per ear, and protein content contributed 68.2%, 11.8%, and 20.0% to protein yield, respectively, with grain weight making the largest contribution, followed by protein content. Enhancing grain weight represents the primary pathway to increasing protein yield.

### 3.2 Effects of nitrogen rate on the accumulation characteristics of soluble and crude proteins in grains of different low-N-tolerant maize hybrids

**Figure 2:**
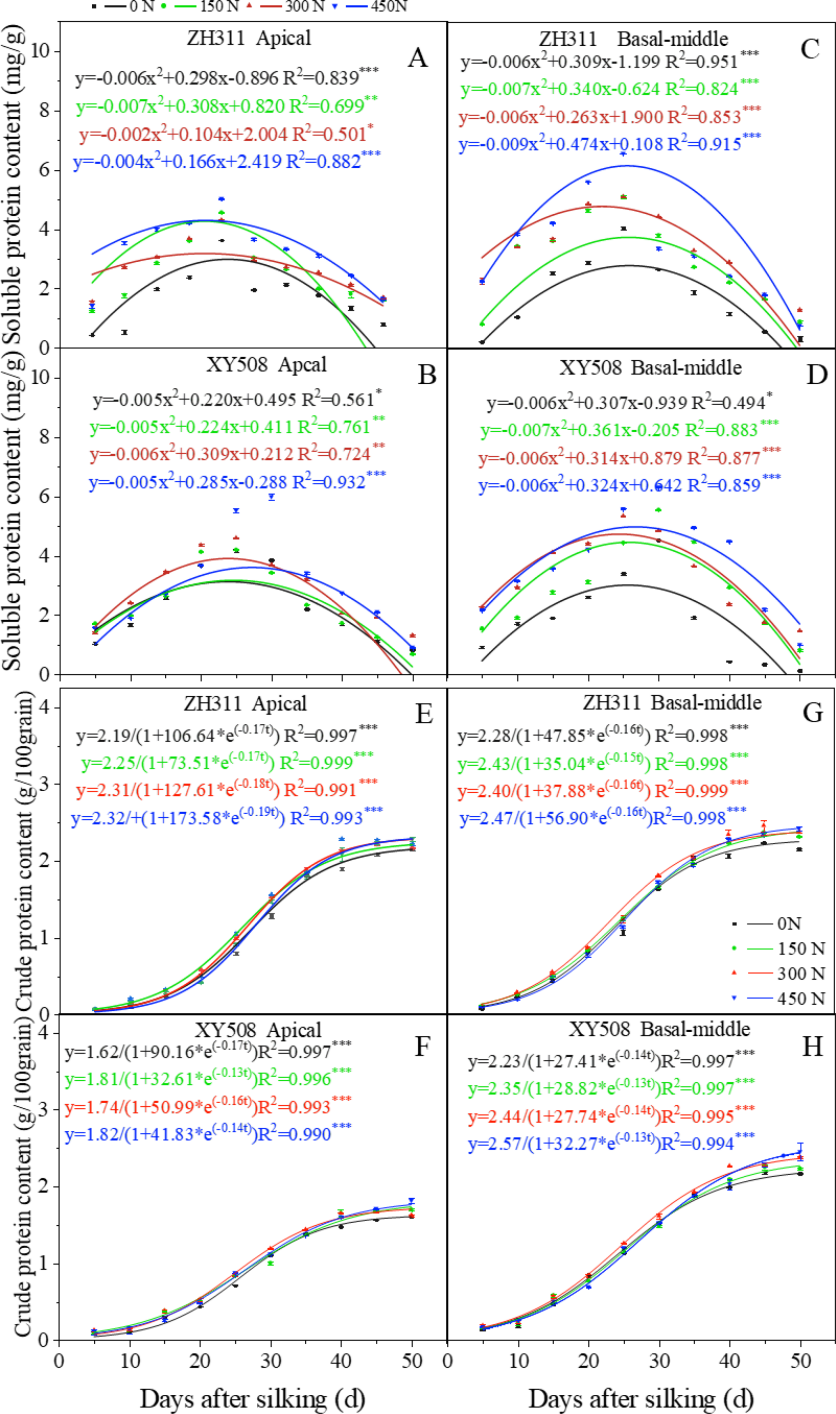
Effects of nitrogen rate on soluble protein content and crude protein accumulation in grains of different low-N-tolerant maize hybrids (average from 2017-2018). **(A–D)** represents soluble protein content (curves are polynomial fits); **(E–H)** represents crude protein accumulation dynamics (curves are logistic fits). The *, **, and *** represent significance levels of P<0.05, P<0.01, and P<0.001, respectively. Vertical bars represented the mean ± SE. (n=6).

After silking, the soluble protein content in maize grains exhibited an initial increase, followed by a subsequent decline, which characterized by a quadratic function (**Figures 2A–D**). In ZH311, the apical and basal-middle grains attained their peak soluble protein content at approximately 23.4 days and 24.6 days after silking, while those in XY508 at around 24.7 days and 26.1 days, respectively. The application of nitrogen fertilizer notably enhanced the soluble protein content in maize grains, particularly in the basal-middle grains of XY508. The timing of maximum soluble protein content in XY508 grains was progressively delayed with increasing nitrogen levels, whereas those in ZH311 were little affected by nitrogen rate.

The crude protein accumulation in maize grains adhered to a logistic growth model (R²=0.990***-0.999***). Different grain positions, cultivars, and nitrogen treatments had different final protein accumulation due to its variation in protein accumulation dynamics (**Figure 2**, **Table 1**). Compared to apical grains, the basal-middle grains exhibited a significantly higher accumulation average rate during the initial increase phase (v_1_), averaging 29.0-50.6% more. They also transition into the rapid increase phase earlier (t_1_) by an average of 1.7-3.6 days and maintain this phase for an extended phase (T_2_), averaging 2.0-2.2 days longer. Consequently, the protein accumulation in basal-middle grains surpasses that of apical grains, particularly in XY508. Average over two years and four nitrogen treatments, the protein accumulation in ZH311 apical grains at the mature stage (averaged at 45 and 50 days after silking) was 5.6% lower than that in basal-middle grains, while in XY508, this reduction reached 26.6%, a statistically significant difference. In comparison to XY508, although ZH311 entered the rapid increase phase later with a relatively shorter duration, its mid- and late-stage average accumulation rates (v_2_ and v_3_) were substantially higher (averaging 16.8%-17.9% and 50.8%-53.0% higher in basal-middle and apical grains, respectively), leading to greater final protein accumulation, particularly in apical grains. On average, protein accumulation at the mature stage in ZH311 basal-middle and apical grains was 2.5% and 31.9% higher, respectively, compared to XY508. Under low-N conditions, protein accumulation of basal-middle and apical grains was 1.0-4.5% and 27.8%-33.6% higher in ZH311 than in XY508.

**Table 1:** Crude protein accumulation characteristic parameters in grains of different low-N-tolerant maize hybrids under different nitrogen levels (average from 2017-2018).

Hybrid	N-level	Apical grain						Basal-middle grain					
		T_1_	T_2_	T_3_	v_1_	v_2_	v_3_	T_1_	T_2_	T_3_	v_1_	v_2_	v_3_
ZH311	0	19.42b	15.26b	18.99b	0.024d	0.083b	0.023b	15.83b	16.34c	20.34b	0.030d	0.081b	0.021c
	150	17.90c	15.82a	19.69a	0.027a	0.082b	0.023b	15.43c	18.14a	22.58a	0.033b	0.077c	0.022b
	300	19.44b	14.49c	18.04c	0.025b	0.092a	0.026a	14.71d	16.72b	22.23a	0.035a	0.083a	0.023a
	450	20.68a	14.19c	17.66c	0.024c	0.095a	0.026a	17.09a	16.52bc	21.96a	0.031c	0.081ab	0.022b
XY508	0	18.21a	15.23d	18.74c	0.019b	0.062a	0.017a	14.56c	18.68b	23.75b	0.032b	0.067c	0.019b
	150	16.43c	19.97a	24.84a	0.023a	0.053d	0.015b	15.39b	19.84a	24.69a	0.032b	0.069b	0.019ab
	300	16.60c	17.09c	21.27b	0.023a	0.060b	0.017a	14.82c	19.56ab	24.22ab	0.035a	0.073a	0.020a
	450	17.12b	18.66b	23.67a	0.022a	0.056c	0.016ab	16.84a	20.56a	25.59a	0.032b	0.072a	0.020a
F-value													
H		**	**	**	**	**	**	**	**	**	**	**	**
N		**	**	**	**	**	**	**	**	**	**	**	**
H×N		**	**	**	**	**	**	**	**	**	*	**	*

T_1_: duration of the initial-increase phase (d); T_2_: duration of the fast-increase phase (d); T_3_: duration of the slight–increase phase (d); v_1_: mean rate of accumulation during the initial-increase phase (g 100grain^-1^ d^-1^); v_2_: mean rate of accumulation during the fast-increase phase (g 100grain^-1^ d^-1^); v_3_: mean rate of accumulation during the slight–increase phase (g 100grain^-1^ d^-1^). * and** represent significant levels of P<0.05 and P<0.01, respectively.

Increasing nitrogen fertilizer could significantly alter the protein accumulation process, thereby affecting the final protein content in grains, particularly in XY508. With increasing nitrogen rate, final protein accumulation in XY508 exhibited a gradual rise, whereas in ZH311, it initially increased then slightly declined. Nitrogen application exerted a more pronounced effect on the apical grains of XY508. The coefficients of variation for ZH311 and XY508 basal-middle and apical grains across four nitrogen levels were 0.041 and 0.028, 0.038, and 0.044, respectively.

**Figure 3:**
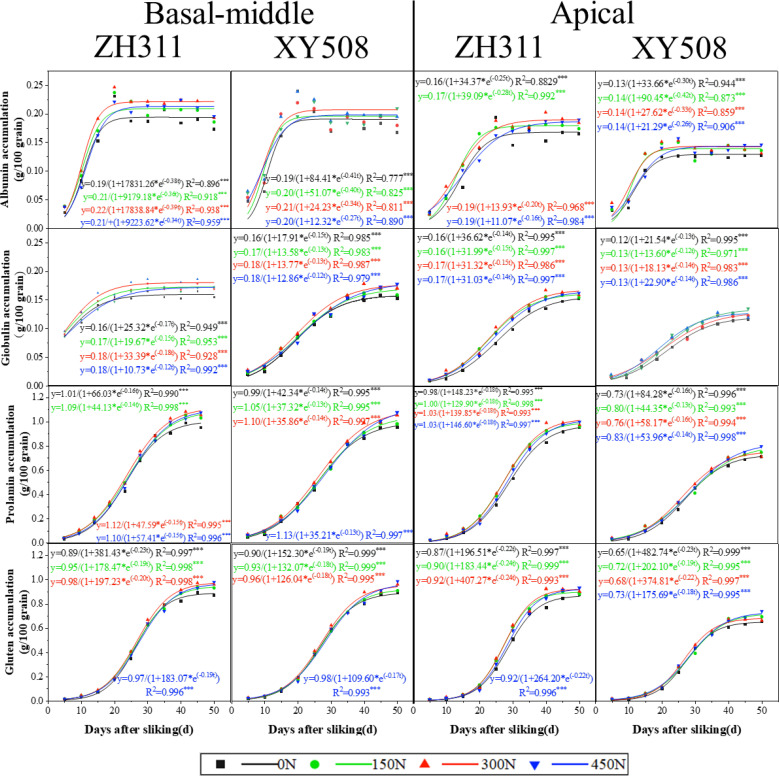
The relationship of protein accumulation with its fractions and accumulation parameters (2017-2018).The correlation coefficients of crude protein accumulation parameters with the theoretical maximum accumulation quantity(a) **(A)** and its contribution rate to a **(B)**; The interaction between protein fraction accumulation and crude protein accumulation **(C, D)** and protein yield **(E, F)**; The correlation coefficients between crude protein accumulation and yield with each protein fraction accumulation **(I)**. T_1_: duration of the initial-increase phase (d); T_2_: duration of the fast-increase phase (d); T_3_: duration of the slight–increase phase (d); v_1_: mean rate of accumulation during the initial-increase phase (g 100grain^-1^ d^-1^); v_2_: mean rate of accumulation during the fast-increase phase (g 100grain^-1^ d^-1^); v_3_: mean rate of accumulation during the slight–increase phase (g 100grain^-1^ d^-1^); Alb, Albumin; Glo, Globulin; Pro, Prolamin; Glu, Gluten; CPA, crude protein accumulation; SPC, soluble protein content; GY, protein yield. *, **, and *** represent significance at P<0.05, P<0.01, and P<0.001. (n=6).

Correlation and path analysis (**Figures 3A, B**) revealed that the theoretical maximum value of protein accumulation (a) in maize grains ware significantly positively correlated with T_1_, v_2_, and v_3_. For apical grains, the most influential factor to a is T_1_, with a contribution rate of 43.04%, followed by v_2_, contributing 23.10%. In basal-middle grains, v_3_ represents the most significant contributor, accounting for 42.44%, followed by T_1_, which contributes 23.89%.

### 3.3 Effects of nitrogen rate on the changes in protein fractions in different low-N-tolerant maize grains

**Figure 4:**
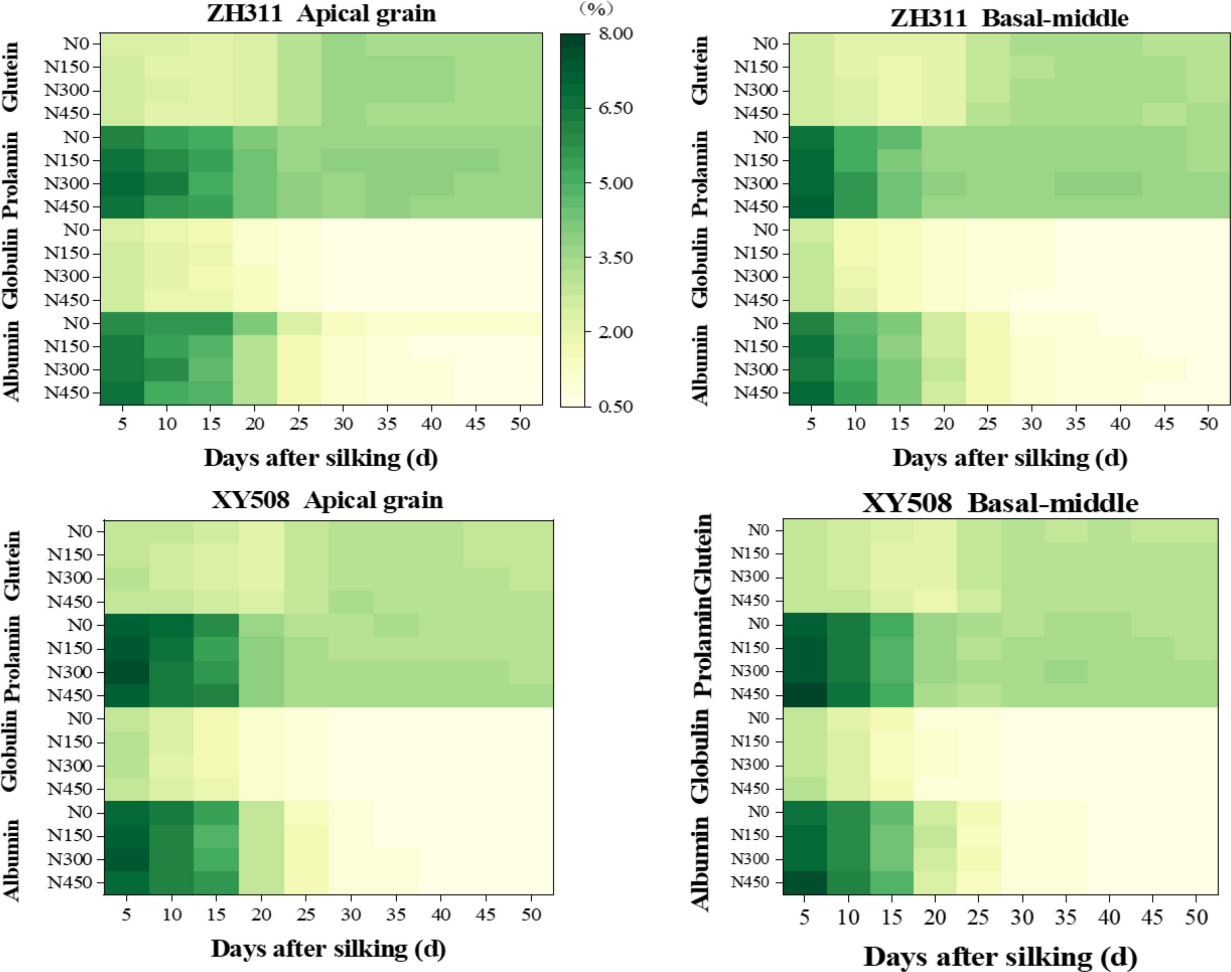
Effects of nitrogen rate on the changes in protein fraction contents in maize grains of different low-N-tolerant hybrids. (n=6).

After silking, the content of albumin, globulin, and prolamin exhibited a decline with either an exponential or reverse logistic function. Conversely, gluten content initially showed a modest reduction, then rapidly increased peaking around 30 DAS (days after silking), followed by a slight decline. In comparison to 5 days after silking, average over two years, two hybrids, and four nitrogen treatments, the content of albumin, globulin, and prolamin at physiological maturity (50 DAS) decreased by approximately 90.1%, 79.9%, and 49.9%, respectively, whereas gluten content exhibited a 19.0% increase (**Figure 4**). After silking, the trends in albumin and globulin proportions were largely consistent with their content, whereas the proportions of prolamin and gluten followed a logistic increase. Prolamin proportions reached stabilization at approximately 40 DAS, whereas gluten stabilized around 30 DAS. At maturity, the proportions of albumin, globulin, prolamin, and gluten were approximately 8.5%, 7.0%, 44.0%, and 40.5%, respectively (**Figure 4**). The content of each protein fraction differed between basal-middle and apical kernels (similar to their crude protein content, with ZH311 showing higher values in apical kernels, while XY508 showed the opposite (Liu et al., 2024), but their proportions had no significant difference.

Increasing nitrogen fertilizer could enhance the content of all protein fractions in both basal-middle and apical kernels at maturity in both hybrids (except for albumin in apical kernels of ZH311), especially in XY508. As nitrogen rate increased, the content of all protein fractions in XY508 showed a gradual increase. In contrast, those in ZH311 exhibited an initial rise, followed by a subsequent decline, particularly in the basal-middle kernels. Overall, nitrogen fertilization exerted a greater influence on the content of albumin and globulin compared to prolamin and gluten. Over two years and across both hybrids, the average coefficients of variation for albumin, globulin, prolamin, and gluten at maturity under four nitrogen rates were 0.030, 0.026, 0.017, and 0.016.

**Figure 5:**
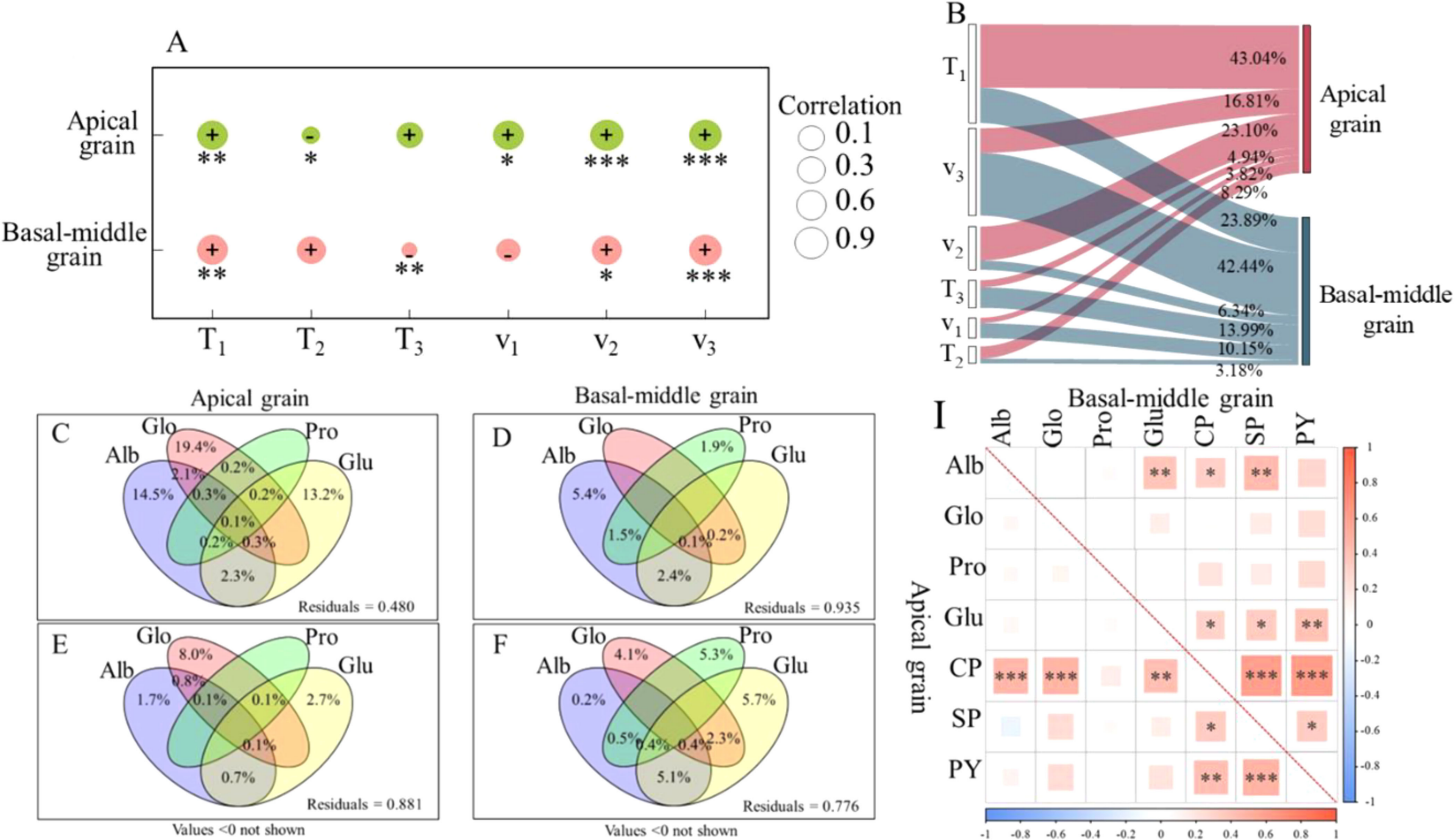
Dynamics of protein fraction accumulation of different low-N-tolerant hybrids after silking (2017-2018 Average). *, **, and *** represent significant levels of P<0.05, P<0.01, and P<0.001, respectively. Vertical bars represented the mean ± SE. (n=6).

After silking, the accumulation of all protein fractions exhibited a progressive increase, conforming to a logistic growth model (**Figure 5**). Albumin exhibited the fastest accumulation, entering the rapid accumulation phase approximately one week and concluding around 17 days after silking (**Table 2**). Globulins, prolamin and glutenin accumulated slowly, especially prolamin. Gluten transitioned into the rapid accumulation phase last with the shortest the fast-increase duration (T_2_) and slight–increase duration (T_3_). In contrast, globulin entered the rapid phase first, exhibiting an extended duration for both phases (T_2_ and T_3_).

**Table 2:** Accumulative characteristic parameters of protein fractions in maize grains of different low-N-tolerant hybrids under different nitrogen rate.

Protein	Hybrid	N-level	Apical						Basal-middle					
fraction			T_1_	T_2_	T_3_	v_1_	v_2_	v_3_	T_1_	T_2_	T_3_	v_1_	v_2_	v_3_
Albumin	ZH311	0	8.90a	10.55c	13.14c	0.0040c	0.0092b	0.0026b	7.23a	6.83b	8.50b	0.0057b	0.0164b	0.0046b
		150	8.31ab	9.32d	11.60d	0.0046b	0.0111a	0.0031a	6.70b	7.29ab	9.07ab	0.0066a	0.0165b	0.0046b
		300	6.58b	13.17b	16.38b	0.0061a	0.0083b	0.0023b	6.77b	6.78b	8.44b	0.0069a	0.0188a	0.0053a
		450	6.84b	16.56a	20.60a	0.0058a	0.0065c	0.0018c	7.59a	7.77a	9.67a	0.0059b	0.0158b	0.0044b
	XY508	0	7.23a	8.66b	10.77b	0.0038c	0.0086b	0.0024c	7.61a	6.42c	8.00b	0.0053c	0.0171a	0.0048a
		150	7.51a	6.20c	7.72c	0.0039c	0.0129a	0.0036a	6.47ab	6.52c	8.11b	0.0064c	0.0173a	0.0049a
		300	6.13c	8.06b	10.04b	0.0049a	0.0102ab	0.0029b	5.55bc	7.81b	9.72b	0.0079b	0.0153b	0.0043b
		450	6.79b	10.27a	12.78a	0.0045b	0.0080b	0.0023c	4.36c	9.62a	11.97a	0.0096a	0.0119c	0.0033c
Globulin	ZH311	0	16.08a	18.55a	23.09a	0.0021b	0.0049c	0.0014a	11.37ab	15.75b	19.47b	0.0030c	0.0059b	0.0017b
		150	14.14c	17.34b	21.58b	0.0024a	0.0054ab	0.0015a	10.75b	16.88ab	21.20ab	0.0034b	0.0059b	0.0017ab
		300	14.31bc	17.72b	22.05b	0.0025a	0.0055a	0.0015a	12.21a	16.71ab	20.79ab	0.0036a	0.0071a	0.0020a
		450	15.00b	18.65a	23.21a	0.0024a	0.0052b	0.0015a	9.15c	18.59b	23.14a	0.0032b	0.0045c	0.0013c
	XY508	0	13.27a	19.93b	24.81b	0.0019c	0.0034b	0.0010b	10.61a	17.82c	22.17c	0.0032b	0.0051a	0.0014a
		150	10.99b	22.38a	27.86a	0.0025a	0.0033b	0.0009b	9.93ab	20.25b	25.20b	0.0036a	0.0049b	0.0014a
		300	11.23b	18.70c	23.28c	0.0024a	0.0039a	0.0011a	9.72b	19.61b	24.40b	0.0039a	0.0052a	0.0015a
		450	13.08a	18.99bc	23.63c	0.0021b	0.0040a	0.0011a	10.33ab	22.01a	27.39a	0.0037a	0.0047b	0.0013b
Prolamin	ZH311	0	21.01a	15.03a	18.71a	0.0098c	0.0375b	0.0105c	17.46b	16.00b	19.92b	0.0122c	0.0362a	0.0102a
		150	19.68b	14.60a	18.18a	0.0108a	0.0396a	0.0111b	17.03b	18.17a	22.61a	0.0135a	0.0347a	0.0097c
		300	20.03b	14.56a	18.12a	0.0109a	0.0409a	0.0115a	16.96b	17.55a	21.85a	0.0139a	0.0368a	0.0103b
		450	20.86a	14.97a	18.62a	0.0104b	0.0395a	0.0111b	17.95a	17.29ab	21.52ab	0.0130b	0.0368a	0.0103b
	XY508	0	19.07a	16.11b	20.05b	0.0081c	0.0262a	0.0073a	16.81b	18.23c	22.69c	0.0125d	0.0316a	0.0088b
		150	18.40b	19.59a	24.38a	0.0093a	0.0239b	0.0067b	17.03b	19.48b	24.25b	0.0131c	0.0313a	0.0088b
		300	17.65c	16.93b	21.07b	0.0091b	0.0260a	0.0073a	16.70b	19.44b	24.19b	0.0140a	0.0327a	0.0092a
		450	19.06a	18.79a	23.39a	0.0092a	0.0255a	0.0072b	17.86a	20.96a	26.08a	0.0134b	0.0312a	0.0087b
Gluten	ZH311	0	22.58a	11.84a	14.73a	0.0081c	0.0424c	0.0119c	20.53a	11.69b	14.54b	0.0092b	0.0440a	0.0123a
		150	21.66b	10.98b	13.67b	0.0088b	0.0472a	0.0132a	20.01b	13.63ab	16.96ab	0.0100a	0.0402a	0.0113c
		300	21.82b	11.10b	13.81b	0.0089a	0.0481a	0.0135a	20.06b	13.32ab	16.57ab	0.0103a	0.0424a	0.0119b
		450	22.47a	11.80a	14.68a	0.0087b	0.0452b	0.0127b	20.52a	13.89a	17.28a	0.0100a	0.0403a	0.0113c
	XY508	0	21.38a	11.58b	14.41b	0.0064d	0.0324ab	0.0091a	19.68b	13.98b	17.40b	0.0096c	0.0370a	0.0104b
		150	21.25b	14.02a	17.45a	0.0072b	0.0297bc	0.0083b	19.87ab	14.67ab	18.26ab	0.0099b	0.0365a	0.0102c
		300	20.66d	12.00b	14.94b	0.0070c	0.0328a	0.0092a	19.66b	14.72ab	18.31ab	0.0103a	0.0377a	0.0106a
		450	21.02c	14.38a	17.89a	0.0074a	0.0294c	0.0082b	20.27a	15.80a	19.66a	0.0102a	0.0357a	0.0100d

T_1_: duration of the initial-increase phase (d); T_2_: duration of the fast-increase phase (d); T_3_: duration of the slight–increase phase (d); v_1_: mean rate of accumulation during the initial-increase phase (g 100grain^-1^ d^-1^); v_2_: mean rate of accumulation during the fast-increase phase (g 100grain^-1^ d^-1^); v_3_: mean rate of accumulation during the slight–increase phase (g 100grain^-1^ d^-1^).

Different lowercase letters indicate significant differences between nitrogen treatments within the same hybrid at the P<0.05 level.

Overall, when compared to basal-middle kernels, the beginning of rapid accumulation phases of albumin and globulin in apical kernels were delayed, with extended durations for each accumulation phase. Prolamin and gluten similarly entered the rapid phase later but exhibited shorter durations for both rapid and slow accumulation phases. However, all four-protein fraction in apical grain accumulated at a slower rate than in basal-middle grains at all phases (except for v_2_ and v_3_ of prolamin and gluten in ZH311). The final differences in each protein fraction accumulation between basal-middle and apical kernels in ZH311 were much lower than in XY508. Average over the two years and four nitrogen treatments, the final accumulation of albumin, globulin, prolamin, and gluten (45 and 50 DAS average) in the apical kernels of ZH311 were respectively 8.8%, 8.1%, 5.9%, and 4.0% lower than that of basal-middle kernels, while those in XY508 were 28.6%, 26.9%, 26.5%, and 26.1% lower, reaching statistically significant difference. The accumulation of all four protein fractions in the basal-middle kernels of ZH311 was on average 2.4% higher than that of XY508, but 31.9% higher in the apical kernels (**Figure 5**).

In comparison to XY508, each protein fraction accumulation in both basal-middle and apical kernels of ZH311 transitioned into the rapid accumulation phase later, with a shorter duration for the rapid and slow accumulation phases (except for albumin in apical kernels). Each protein fraction accumulation rate at all phases in apical kernels (except for albumin) and at fast & slow increase phases (T_2_ and T_3_) in basal-middle kernels of ZH311 were slowly than those of XY508. Nitrogen fertilizer also had a certain effect on the accumulation dynamics of each protein fraction and consequently on its final accumulation quantities, but the trend and degree of these effects were different for the two hybrids. As the nitrogen rate increased, the final accumulation of each protein fraction in basal-middle and apical grains of ZH311 (45 and 50 DAS average).

### 3.4 Analysis of factors affecting protein accumulation in maize grain

The results of correlation analysis indicated that crude protein accumulation quantity (CPA) in apical grains exhibited a significant positive correlation with the accumulation of Alb, Glo, and Glu, with correlation coefficients of 0.47***, 0.49***, and 0.43**, respectively (**Figure 3I**). Furthermore, Protein yield (PY) was strongly and significantly positively correlated with both CPA and soluble protein content (SPC), with correlation coefficients of 0.43** and 0.49***. CPA in basal-middle grains demonstrated a significant correlation with the accumulation of Alb and Glu (0.32* and 0.30*), while PY exhibited significant or highly significant positive correlations with Glu, CPA, and SPC (0.40**, 0.62***, and 0.34*).

VPA and correlation analysis revealed that the combined interaction of Alb, Glo, Pro, and Glu accumulation accounted for 52.0% and 6.5% of the crude protein accumulation in apical and basal-middle grains, respectively, and contributed 11.9% and 22.4% to the protein yield in apical and basal-middle grains, respectively. Glo accumulation emerged as the primary factor influencing crude protein accumulation and protein yield in apical grains, contributing 19.4% and 8.0%, respectively. The contribution rates of Alb and Glu accumulation to crude protein in apical grains were relatively elevated, at 14.5% and 13.2%, respectively (**Figures 3 C, E**). The predominant factors influencing crude protein accumulation and protein yield in basal-middle grains were the accumulations of Alb and Glu, with contribution rates of 5.4% and 5.7%, respectively (**Figures 3 D, F**). These findings were consistent with those of the correlation analysis.

**Figure 6:**
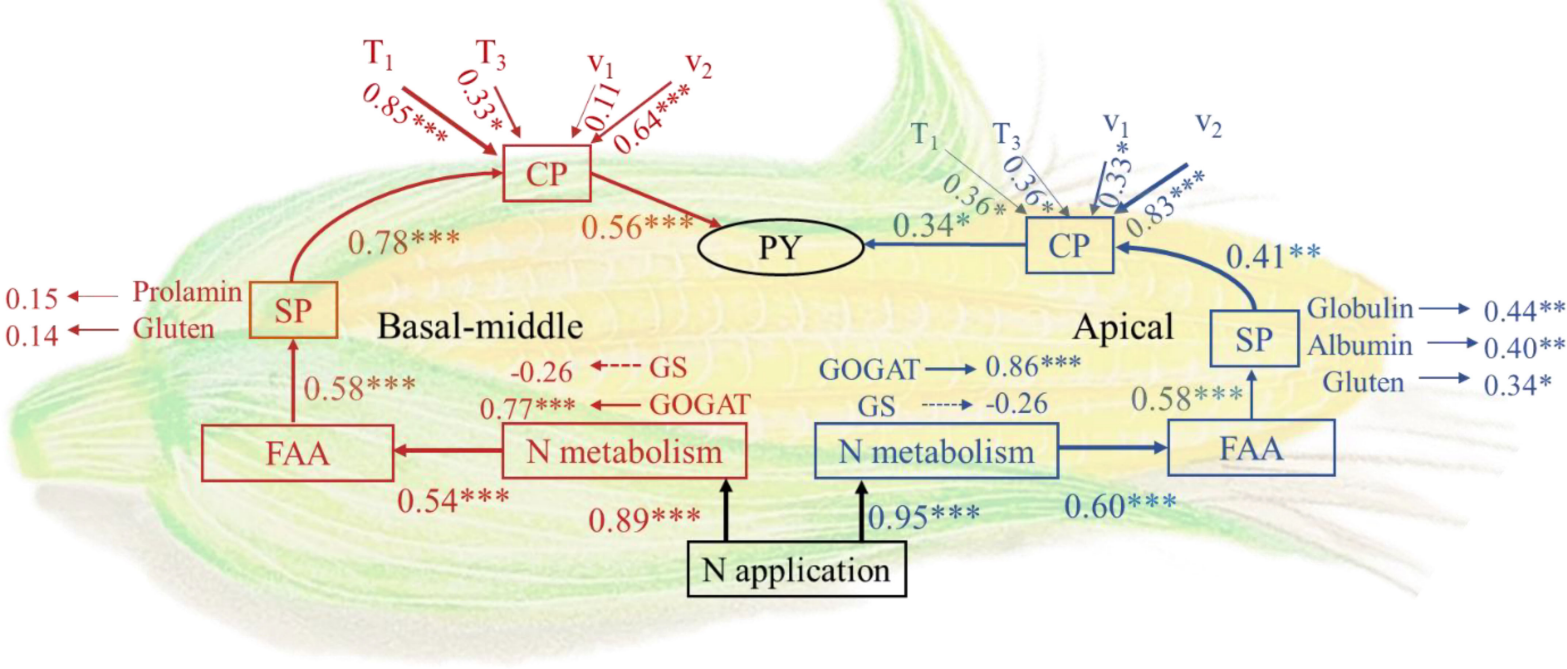
Structural equation model (SEM). GS, Glutamine synthetase; GOGAT, Glutamate synthase; FAA, Free amino acids; SPC, Soluble protein content; CPA, Crude protein accumulation; PY, Protein yield; T_1_: duration of the initial-increase phase (d); T_2_: duration of the fast-increase phase (d); T_3_: duration of the slight–increase phase (d); v_1_: mean rate of accumulation during the initial-increase phase; v_2_: mean rate of accumulation during the fast-increase phase; v_3_: mean rate of accumulation during the slight–increase phase. Red represents basal-middle grains, and blue represents apical grains. Single arrows indicate the hypothesized direction of causal relationships. Indicators represent standardized path coefficients showing positive (positive values) or negative (negative values) effects. Solid lines represent positive paths, and dashed lines represent negative paths. The width of the arrows indicates the strength of the causal relationship. The data of GS, GOGAT, and FAA can be found in Liu et al. (2024) and Wu et al. (2022). *, **, and *** indicate P<0.05, P<0.01, and P<0.001, respectively. (n=6).

Structural equation modelling (SEM) analysis was employed to investigate the formation process of protein yield at different grain positions in maize. Nitrogen fertilizer application modulates protein accumulation by influencing nitrogen metabolism in maize grains, thus impacting protein yield. Nitrogen fertilizer exerted a highly significant positive effect on enzyme activities associated with nitrogen metabolism, especially the GOGAT which directly and significantly facilitated FAA formation. The influence of nitrogen fertilizer on protein metabolism in apical grains was more pronounced than in basal-middle grains. Throughout the protein accumulation process, the accumulation means rate of accumulation during the fast-increase phase(v_2_) and duration of the initial-increase phase(T_1_) exerted a more substantial influence on protein accumulation. Enhancing the accumulation v_1_ and appropriately extending T_1_ could effectively promote protein accumulation. Moreover, the accumulation v_3_ also significantly influenced protein accumulation in apical grains (**Figure 6**).

## 4 Discussion

### 4.1 Differences in protein accumulation between basal-middle and apical grains in different low-N-tolerant maize hybrids

Nitrogen application enhanced the activity of enzymes associated with nitrogen metabolism (Liu et al., 2024), which process promotes protein synthesis and accumulation (Su et al., 2022), thereby enhancing the content (Wu et al., 2022; Liu et al., 2024) and yield of protein in grains (Jawad et al., 2017). However, our findings demonstrated that these effects varied significantly between maize hybrids with different low-N-tolerance and across different grain positions. Prior research had indicated that during the grain-filling stage in cereal crops, assimilates were preferentially allocated to basal-middle grains, leading to an insufficient supply to apical grains and resulting reduction of its grain weight (Xu et al., 2018; Luo et al., 2021; Wu et al., 2022). In wheat, apical grains exhibit lower protein content than basal-middle grains (Zhu et al., 2022), whereas the opposite trend was observed in rice (Zhang et al., 2020; Liu et al., 2022). In this study, the protein content in apical grains of XY508 was marginally lower than that in basal-middle grains, whereas in ZH311, it surpassed that of basal-middle grains. However, due to the lower grain weight of apical grains in both hybrids, their protein accumulation quantity and yield were markedly lower than those of basal-middle grains. This phenomenon was especially pronounced in the low-N-sensitive hybrid XY508 and the protein yield. In contrast to XY508, the difference in protein accumulation between basal-middle and apical grains was less pronounced in the low-N-tolerant hybrid ZH311, especially under low-N conditions. This observation may help explain the enhanced protein yield of this hybrid. The effect of nitrogen fertilizer on increasing protein accumulation and yield in XY508 was greater than in ZH311, especially for apical grains, and the difference between the two hybrids diminished as nitrogen rate increased (**Figure 2**). This indicated that similar to grain yield, the advantage in protein yield observed in the low-N-tolerant hybrid ZH311 under low-N conditions was more pronounced. This finding could be attributed to ZH311’s stronger nitrogen absorption capacity (Li et al., 2016), slower leaf senescence, higher photosynthetic capacity under low-N conditions (Wu et al., 2019), improved grain filling (Wu et al., 2022), more vigorous nitrogen metabolism, and higher protein content (Liu et al., 2024). These results provided a theoretical basis for the development of nitrogen reduction and efficiency enhancement strategies tailored to specific hybrids.

The protein accumulation process in maize grains after silking adheres to a logistic equation, progressed through three stages: the initial-increase phase (T_1_), the fast-increase phase (T_2_), and the slight–increase phase (T_3_). Overall, the protein accumulation process occurred at a slightly faster than the grain filling process (i.e., grain weight increase process), with each stage averaging approximately 1-2 days shorter (Li et al., 2020). It indicated that nitrogen metabolism was more vigorous than carbon metabolism during the early grain-filling period. Nevertheless, this phenomenon exhibited notable variations among grain positions, maize hybrids, and nitrogen rates. In general, apical grains exhibited a longer T_1_, a shorter T_2_, and a lower v_1_ than basal-middle grains. When compared to XY508, ZH311 demonstrated a longer T_1_, a shorter T_2_, but a higher v_2_ and v_3_, especially in apical grains. Moderate nitrogen application could enhance parameters such as v_1_ and T_1_, thereby promoting protein accumulation, particularly in XY508 and apical grains. Previous studies have demonstrated that the primary parameters influencing grain weight in rice were v_2_ and T_1_ for apical grains and T_2_ for basal-middle grains (Wen et al., 2022), while those in wheat were T_1_, v_2_, and v_3_ (Wu et al., 2014), and in maize were T_2_, v_2_, or T_3_ (Li et al., 2020). Although T_1_ played a less role in maize grain weight, it contributed more to protein accumulation (in addition, the v_2_ for apical grains and v_3_ for basal-middle grains contributed larger too). This phenomenon might be attributed to the earlier and faster process of protein accumulation compared to grain weight, suggesting that entering the rapid growth phase prematurely does not substantially enhance protein accumulation. Enhancing the accumulation rate during the mid to late stages (the rapid increase to slight increase phase) represents a crucial technical strategy for augmenting protein accumulation.

### 4.2 Dynamic changes in protein fractions and the effects of nitrogen fertilizer

After cereals mature, the primary proteins in grains are storage proteins. For rice, glutelin is the main storage protein, accounting for around 70%, with a glutelin-to-prolamin ratio of 5-12 (Lu et al., 2022). Wheat has a lower ratio of about 1.1 (Yang et al., 2007). In maize, the content of prolamin was higher than that of gluten, with about 44% prolamin and 40% gluten (**Figure 5**), which is similar to the results of previous studies (Fu, 2020) (**Figure 5**).

In terms of percentage by weight, after flowering, albumin and globulin contents in wheat grains gradually decrease, while glutelin and prolamin contents gradually increase (Zhao et al., 2016). All four protein fractions content in rice increase steadily (Lan et al., 2019). This study showed that albumin, globulin, and prolamin content in maize decreased rapidly by 90-50%, while glutelin content slightly decreased at first, then increased quickly. However, regarding the accumulation quantity, all four protein fractions in the three major cereals consistently increase, with maize exhibiting a logistic accumulation pattern (**Figure 3**). Among these, albumin entered the rapid accumulation phase first, with a shortest duration, and accumulation process much more rapidly than the grain filling (Li et al., 2020). Prolamin and glutelin entered the rapid accumulation phase later, but accumulated faster (especially the fast and slight accumulation phase), while globulin accumulated the slowest. Compared to basal-middle kernels, all protein fractions in apical kernels entered the rapid accumulation phase later, with a longer rapid and slight accumulation phases for albumin and shorter ones for prolamin and glutelin. Compared to ZH311, the protein fractions in XY508 entered the rapid accumulation earlier, but accumulated more slowly in each phase (except for albumin during both rapid and slight accumulation phases in apical kernels) (**Table 2**). These results provide theoretical support for timely regulation strategies to increase the accumulation of specific protein fraction.

The accumulation of each protein fraction was influenced not only by genotype and grain position but also by nitrogen fertilizer management strategies. It is widely recognized that increasing nitrogen fertilizer can enhance the content and accumulation of protein fractions in cereals (Yang et al., 2009), however, excessive nitrogen might lead to decrease in their levels (Li and Li, 1995). This study showed that nitrogen fertilizer influenced the final content and accumulation of each protein fraction by affecting their accumulation parameters. The final content and accumulation of each protein fraction of XY508 were the highest at 450N, while that of ZH311 300N, which were much higher under 0-150N than XY508. For protein fractions, most studies suggested that nitrogen fertilizer has a greater impact on storage proteins in wheat and rice (Han et al., 2021; Wu et al., 2022), though some argue the differences in effect on them are insignificant (Dai et al., 2019). Shi et al. (2005) found that as the timing of nitrogen application in wheat is delayed, albumin and globulin content decreased, while prolamin and glutelin content increased. This study shows that nitrogen fertilizer did not significantly differ its effect on the proportion of each protein fractions maize. The variation in albumin and globulin content among nitrogen rates was slightly greater than that of prolamin and glutelin. Whether this is a characteristic of maize hybrids or related to experimental conditions and fertilization timing needs, along with the physiological and molecular mechanisms by which nitrogen affects maize protein fractions, to be further studied.

## 5 Conclusion

After silking, the accumulation of protein and its fractions in maize grains increase as a Logistic function, and the accumulation parameters and final accumulation quantities varied not only by protein fractions, but also by genotype, grain position and nitrogen rate. The accumulation of albumin was the fastest, and prolamin and glutenin accumulated more. The accumulation of protein and its fractions in apical grains were less than basal-middle grains and these difference in low-N-tolerant hybrid ZH311 were much smaller than low-N-sensitive hybrid XY508. Nitrogen fertilizer had a greater effect on the protein accumulation and yield of XY508, and ZH311 had a higher accumulation quantity and yield of protein and its fractions under low-nitrogen condition, especially in apical grains. How to increasing the protein accumulation of apical grains, especially under low-N conditions, is an important goal of high-quality maize hybrid breeding and management strategies developing.

## Data Availability

The original contributions presented in the study are included in the article/supplementary material. Further inquiries can be directed to the corresponding author.
