# Supplementary material for: Effects of nitrogen fertilizer on protein accumulation in basal-middle and apical kernels of different low nitrogen tolerant maize hybrids
Source: Front Plant Sci. 2025 Feb 21;16:1526026. doi: 10.3389/fpls.2025.1526026 (PMC11885128; doi:10.3389/fpls.2025.1526026)
Supplement: Supplementary file 1 [file DataSheet1.docx]

**TableA.1** Baseline soil fertility in the 0-20 cm layer before maize sowing in 2017-2018.

| Year | Total N  (g/kg) | Organic matter  (g/kg) | Alkaline N  (mg/kg) | Available P  (mg/kg) | Available K  (mg/kg) | pH |
| --- | --- | --- | --- | --- | --- | --- |
| 2017 | 1.55 | 10.32 | 110.6 | 11.16 | 131.91 | 5.78 |
| 2018 | 1.51 | 11.13 | 108.53 | 15.43 | 105.27 | 5.90 |

Note: N: nitrogen; P: phosphorus; K: potassium.

**TableA.2** 100 kernel weight, number of grains in a spike and nitrogen content of grains of different grain positions of the two varieties (average 2017-2018)

|  |  | Total | | |  | Apical | |  | Basal-middle | |
| --- | --- | --- | --- | --- | --- | --- | --- | --- | --- | --- |
| Hybrid | N-level | 100 kernel weight(g) | Kernels per ear | protein content(%) |  | 100 kernel weight(g) | protein content(%) |  | 100 kernel weight(g) | protein content(%) |
| ZH311 | 0N | 30.87b | 544.39ab | 8.01abc |  | 26.69b | 8.09c |  | 29.18f | 7.44bde |
|  | 150N | 31.82ab | 555.27a | 8.08ab |  | 26.65b | 8.16b |  | 29.64e | 7.82bc |
|  | 300N | 32.92a | 542.98ab | 8.43a |  | 26.69b | 8.32a |  | 29.71e | 8.00ab |
|  | 450N | 32.98a | 518.49ab | 8.22ab |  | 27.43a | 8.33a |  | 29.92d | 8.09a |
|  |  |  |  |  |  |  |  |  |  |  |
| XY508 | 0N | 28.73c | 501.26b | 7.59bc |  | 22.53e | 7.18f |  | 30.27c | 7.17f |
|  | 150N | 31.06b | 518.24ab | 7.95abc |  | 23.73d | 7.17f |  | 30.32c | 7.36ef |
|  | 300N | 32.03ab | 569.37a | 7.86abc |  | 22.31e | 7.28e |  | 31.74b | 7.54de |
|  | 450N | 32.65a | 535.13ab | 7.39c |  | 24.25c | 7.50d |  | 31.97a | 7.63cd |
|  | F-value |  |  |  |  |  |  |  |  |  |
|  | H | ** | ns | ** |  | ** | ** |  | ** | ** |
|  | N | ** | ns | ns |  | ** | ** |  | ** | ** |
|  | H*N | ns | ns | ns |  | ** | ** |  | ** | ns |

Different lower case letters represent significant differences between different nitrogen fertiliser treatments of the same variety at the P<0.05 level. The symbol *, indicated significant at P<0.05. **, significant at P<0.01. NS, not significant.

**TableA.3** F-value test for seed protein fractions at different grain positions.

| protein |  | |  |  | Apical | | | | | |  | Basal-middle | | | | | |
| --- | --- | --- | --- | --- | --- | --- | --- | --- | --- | --- | --- | --- | --- | --- | --- | --- | --- |
| component |  |  |  |  | T_1_ | T_2_ | T_3_ | v_1_ | v_2_ | v_3_ |  | T_1_ | T_2_ | T_3_ | v_1_ | v_2_ | v_3_ |
| F-value | | |  |  | | | | | | | | | | | | | |
| Albumin |  | H | | | * | ** | ** | ns | ns | ns |  | ** | ns | ns | ** | ns | ns |
|  |  | N | | | * | ** | ** | ns | ** | ** |  | ** | ** | ** | ** | * | * |
|  |  | H×N | | | ns | ns | ns | ns | ** | ** |  | ** | * | * | ** | ns | ns |
| Globulin |  | H | | | ** | ** | ** | ** | ** | ** |  | ** | ** | ** | ** | ** | ** |
|  |  | N | | | ** | ** | ** | ** | ** | ** |  | ** | ** | ** | ** | * | ** |
|  |  | H×N | | | ns | ** | ** | ns | * | ** |  | ns | ns | ns | ns | ns | ns |
| Prolamin |  | H | | | ** | ** | ** | ** | ** | ** |  | * | ** | ** | ns | ** | ** |
|  |  | N | | | ** | ** | ** | ** | ** | ** |  | ** | ** | ** | ** | * | ** |
|  |  | H×N | | | ns | ** | ** | ns | ** | ** |  | ns | ** | ** | ** | ns | * |
| Gluten |  | H | | | ** | ** | ** | ** | ** | ** |  | ** | ** | ** | * | ** | ** |
|  |  | N | | | ** | ** | ** | ** | ** | ** |  | ** | * | * | ** | ns | ns |
|  |  | H×N | | | ** | ** | ** | ** | ** | ** |  | * | ns | ns | * | ns | ns |

ns,* and** represent significant levels of not significant, P<0.05 and P<0.01, respectively.

**Fig.A.1** Average temperature and precipitation during the test period 2017-2018.

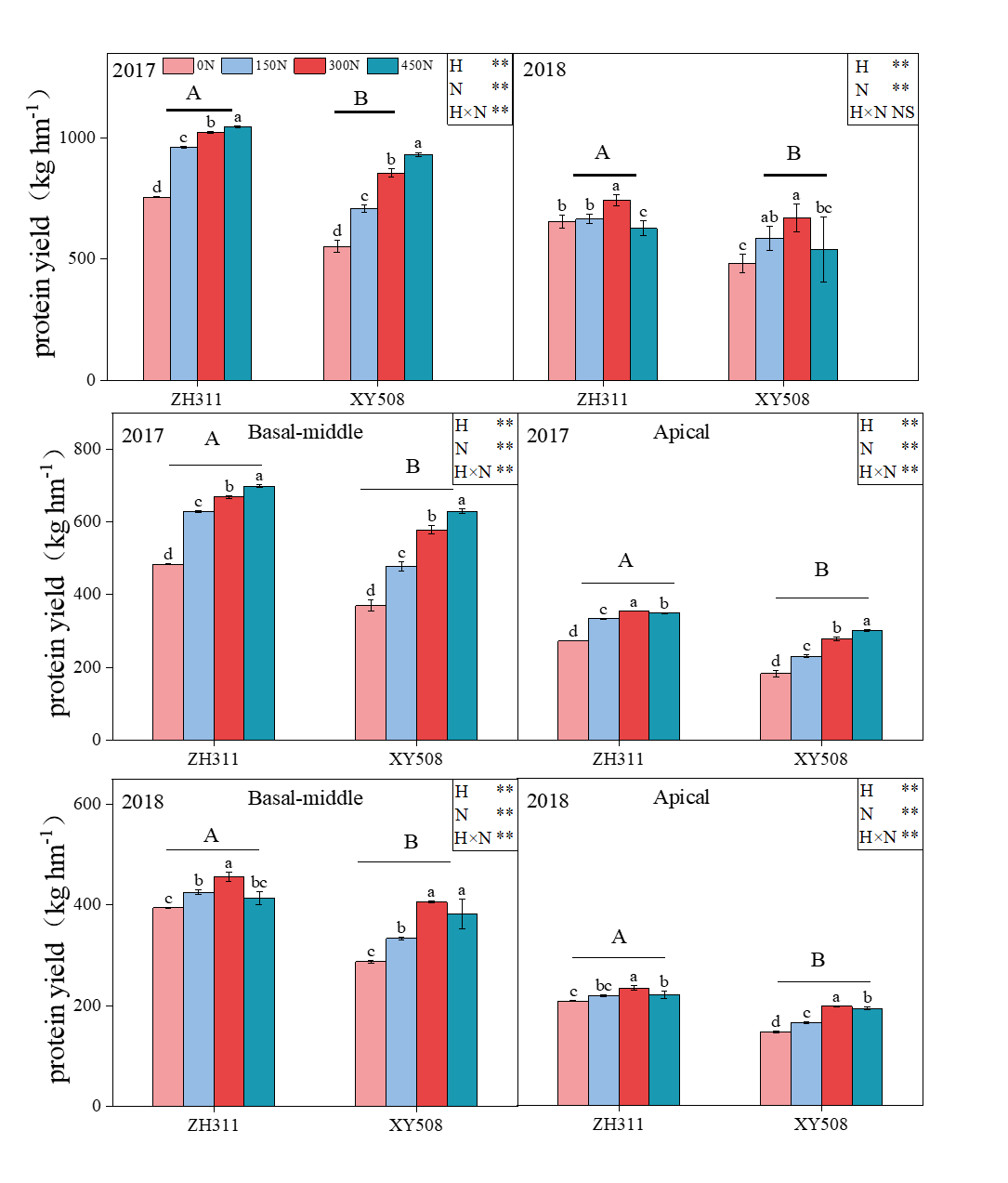


**Fig.A.2** Effects of nitrogen fertilizer rate on protein yield of different low-N-tolerant maize hybrids. The symbol *, indicated significant at P<0.05. **, significant at P<0.01. NS, not significant


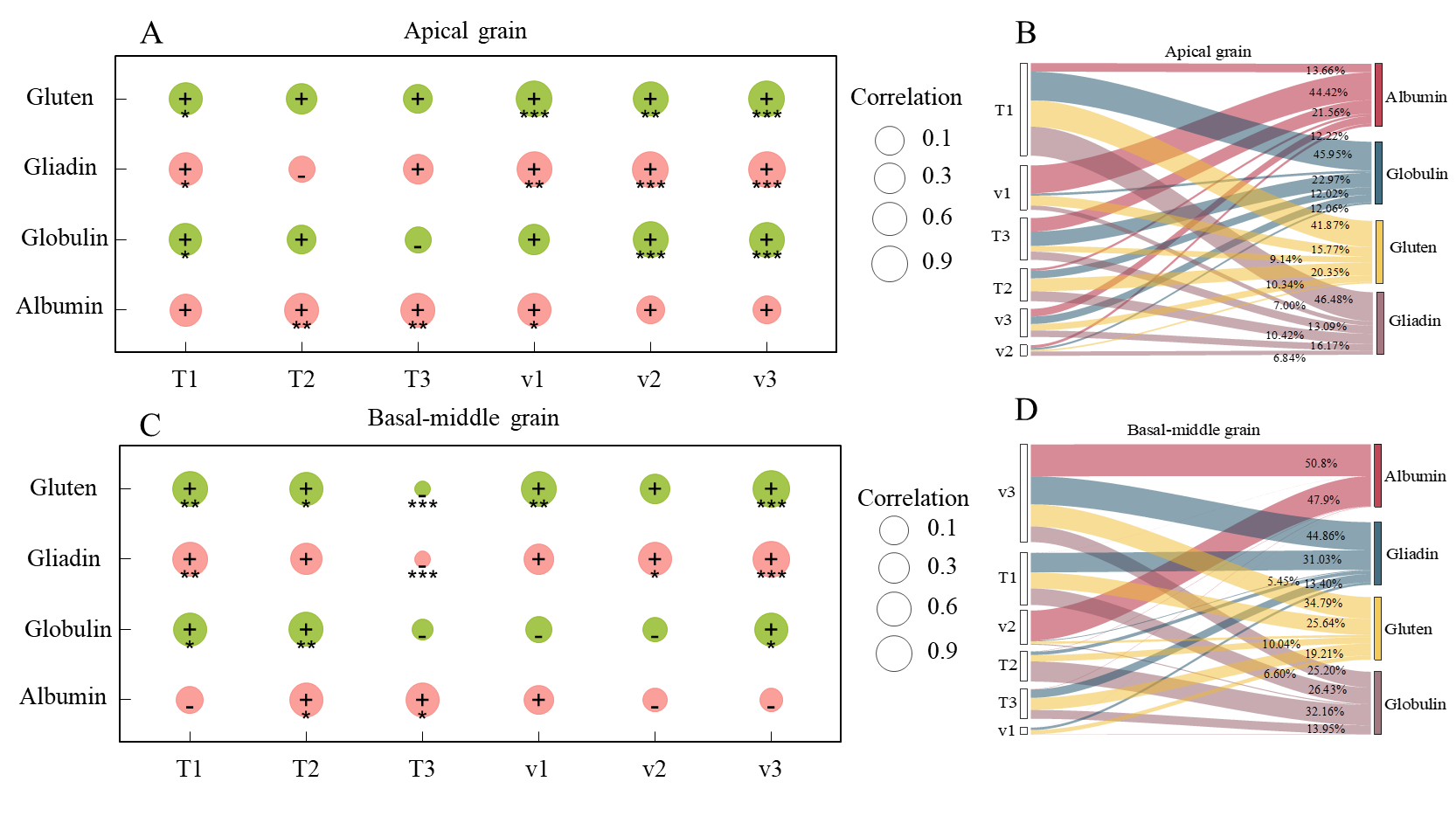


**Fig.A.3** Effect of individual parameters of protein fractions on the accumulation of each protein (a). *, **, and *** represent significant levels of P<0.05, P<0.01, and P<0.001, respectively.
